# Juvenile psammomatoid ossifying fibroma with aneurysmal bone cyst in the posterior mandible

**DOI:** 10.3332/ecancer.2014.471

**Published:** 2014-10-14

**Authors:** Sandhya Tamgadge, Tamgadge Avinash, Sudhir Bhalerao, Sonali Rajhans

**Affiliations:** 1 Prof & PG Guide Dept of Oral & Maxillofacial Pathology and Microbiology Padmashree, Dr D Y Patil Dental College & Hospital, Sector 7, Nerul, Navi Mumbai, Maharashtra 400706, India; 2 Prof & HOD Dept of Oral & Maxillofacial Pathology and Microbiology Padmashree, Dr D Y Patil Dental College & Hospital, Sector 7, Nerul, Navi Mumbai, Maharashtra 400706, India; 3 Prof & PG Guide Dept of Oral & Maxillofacial Pathology and Microbiology Padmashree, Dr D Y Patil Dental College & Hospital, Sector 7, Nerul, Navi Mumbai, Maharashtra 400706, India; 4 PG Student Department of Oral & Maxillofacial Pathology and Microbiology Padmashree, Dr D Y Patil Dental College & Hospital, Sector 7, Nerul, Navi Mumbai, Maharashtra 400706, India

**Keywords:** aneurysmal bone cyst, fine needle aspiration cytology (FNAC), mandible, psammomatoid juvenile ossifying fibroma, psammoma bodies

## Abstract

Aneurysmal bone cysts (ABCs) are a rare benign lesion seen as locally destructive, rapidly expansile, and mostly affecting the long bones and vertebrae. The association of ABCs with juvenile psammomatoid ossifying fibroma (PsJOF) is predominantly seen in the extragnathic region, and it is extremely rare with only a few cases reported so far in the mandible. Here, we report one such case of a hybrid lesion in a seven-year-old boy, who presented with a solitary swelling of the left mandible showing partial obliteration of buccal vestibular sulcus, which shows juvenile psammomatoid ossifying fibroma as a pre-exsiting lesion, transforming into an ABC. Such hybrid lesions are usually misdiagnosed and have been sparsely reported in the dental literature.

## Introduction

All fibro-osseous lesions consist of the replacement of normal bone architecture with benign fibrous tissues composed of fibroblast and collagen. They also show varying amounts of mineralised material. This includes a broad group of several entities such as ossifying fibroma, juvenile ossifying fibroma, fibrous dysplasia, and so on [1]. Out of these, juvenile ossifying fibroma (JOF) is a benign bone-forming true neoplasm, and it is defined as a variant of the ossifying fibroma in the extragnathic craniofacial skeleton of young patients [2, 3]. JOF commonly occurs in the facial bones (85%), calvarium (12%), and mandibular region (10%). Very rarely it has been reported extracranially (3%) [4]. JOF has two subtypes: psammomatoid ossifying fibroma and trabecular ossifying fibroma [5, 6] of which the trabecular type commonly involves the jaws [7]. Juvenile psammomatoid ossifying fibroma (PsJOF) are unique lesions that occur commonly in children. Psammoma-like bodies are the hallmark of this neoplasm. An aneurysmal bone cyst (ABC) can occur as a secondary change in association with a number of benign and malignant bone lesions [8]. Mandibular lesions are uncommon and can be mistaken for an odontogenic cyst [9]. To the best of our knowledge, the number of PsJOF cases converting into ABC have not been reported in the literature so far, but Makek in his study found out that of the 69 cases of PsJOF, only three cases showed ABC transformation. Based on this study the estimated percentage would be 4.3% [10]. Here, we report an additional case of PsJOF that occurred in the mandible, an uncommon site, and was associated with ABC, which is also a rare entity.

## Case report

**Figure 1.**
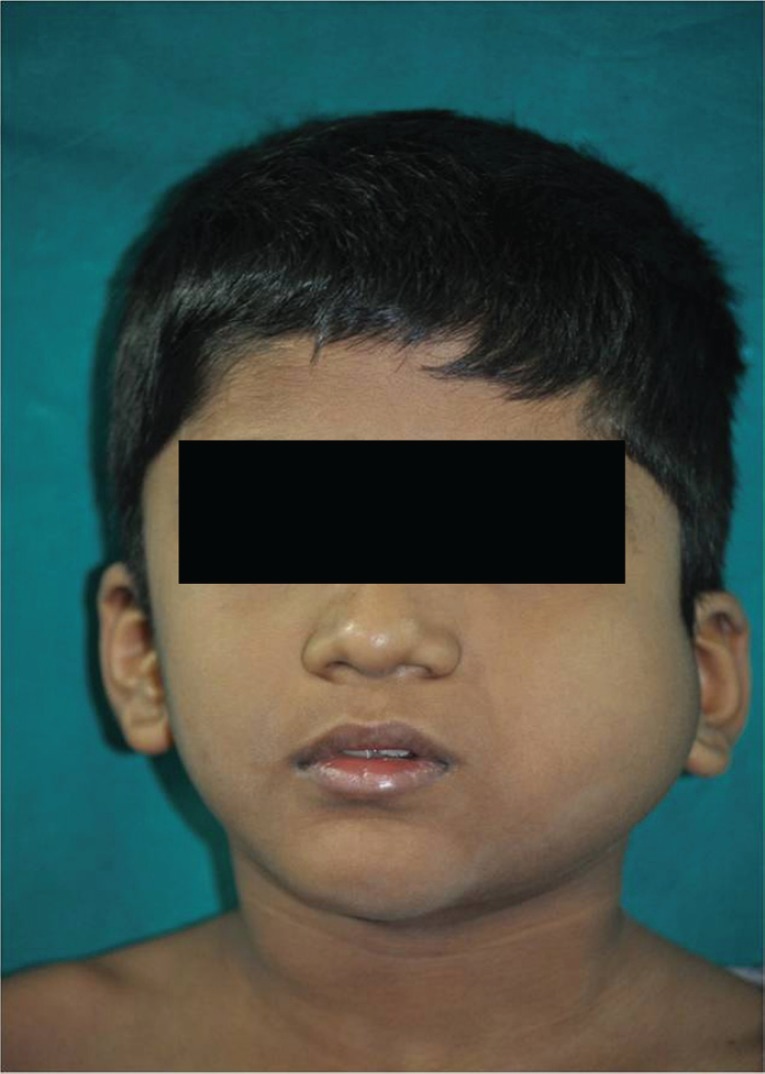
Swelling on left side of face extending from zygomatic arch to lower border of mandible superoinferiorly. Swelling was covering the whole ramus of the mandible anteroposteriorly.

**Figure 2.**
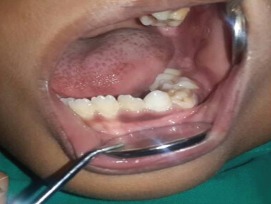
Intraoral examination shows obliteration of buccal sulcus and displacement of first and second deciduous molar and first permanent molar.

**Figure 3.**
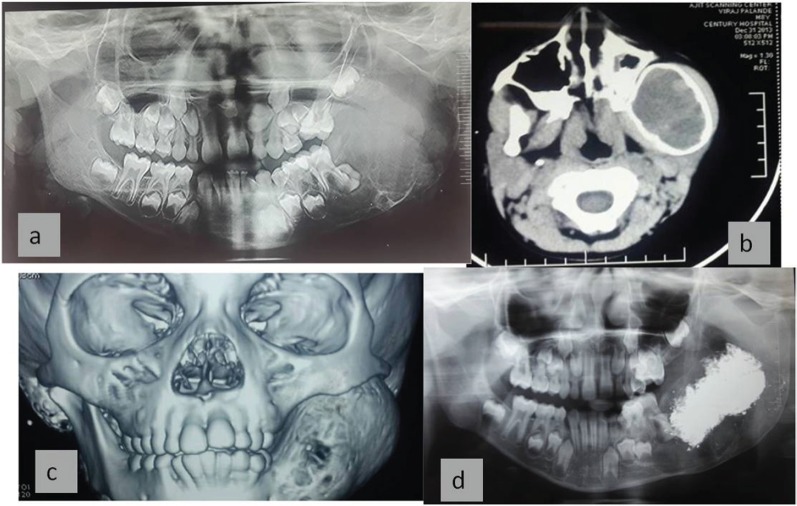
X-ray and CT scan show (a) large multilocular mixed radiolucent radiopaque lesion involving left side of mandible extending from mandibular, second deciduous molar. Superiorly it extends up to condylar and coronoid processes. Inferior margin showed multiple septae with thinning of the cortical plates, Anterior displacement of the tooth bud 37 was noted. (b) The CT sections show large severely expansile lesion in the left ramus of the mandible extending up to the cortical plates. (c) 3D CT shows expansion and thinning of cortical plates localised areas with perforation of cortical plates noted. (d) shows postoperative follow-up after two months with bony healing inferiorly.

**Figure 4.**
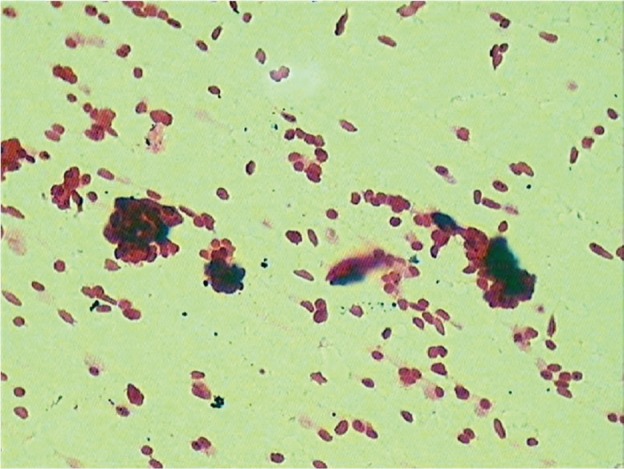
Cytological smears show spindle-shaped stroma with giant cells (40x).

**Figure 5.**
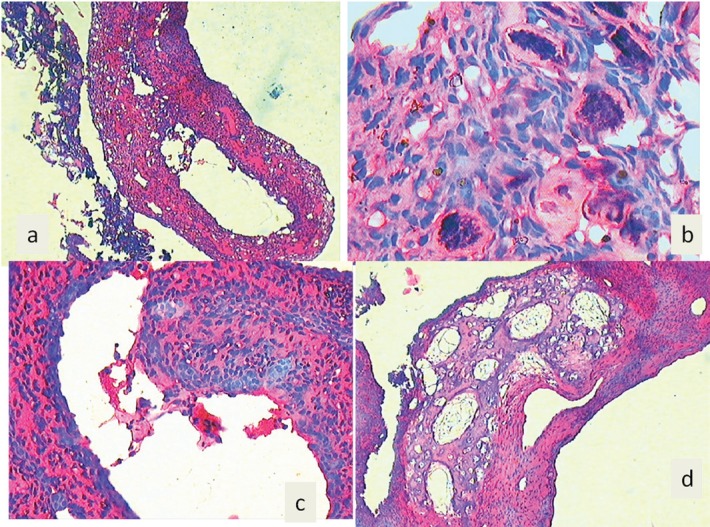
H&E stained section shows (a) both lesions, i.e., ABC and JOF, (b) psammoma bodies under higher magnification, (c) giant cells lining the sinusoidal spaces, (d) new bone formation at the periphery.

**Figure 6.**
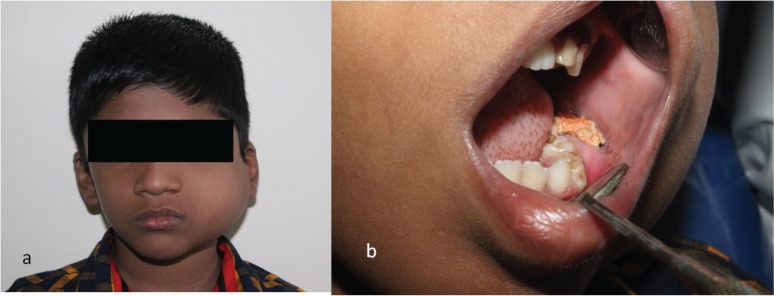
Postoperative follow-up after two months (a) extraoral (b) intraoral.

A seven-year-old boy complained of a gradually increasing swelling on the left side of his face for the past year. Initially the swelling was small but gradually increased in size during the past six months. On extraoral examination, the swelling extended from the zygomatic arch to the lower border of the mandible superoinferiorly. The swelling was covering the whole ramus of mandible anteroposteriorly (Figure1). On palpation it was bony hard and there was obliteration of buccal sulcus. Intraorally there was expansion of both buccal and lingual cortical plates and displacement of first and second deciduous molar and first permanent molar (Figure 2). Radiographically, a large multilocular, mixed radiolucent radiopaque lesion was seen on the left side of the mandible extending from the mandibular second deciduous molar up to the posterior border of the ramus. Superiorly, it extended up to the condylar and coronoid processes. The inferior margin showed multiple septae with thinning of the cortical plates. Anterior displacement of the tooth bud 37 was noted. Based on the preliminary findings of an orthopantomogram (OPG), the lesion looked like a benign odontogenic tumour like ameloblastoma, non-odontogenic tumours like JOF, ossifying fibroma, ABC. The computed tomography (CT) sections showed a large severely expansile lesion in the left ramus of the mandible extending up to the cortical plates. Superiorly, it was extending up to the sigmoid notch, the coronoid process, and the ramus. The interior of the lesion showed a heterogenously enhancing soft tissue mass with multiple bony septae. The inferior extent of the lesion showed hyperdense septae (Figure 3). FNAC (Figure 4) showed blood-tinged fluid. Microscopic examination showed moderate cellularity with oval to spindle-shaped cells along with numerous multinucleated giant cells.Treatment options were as follows: enucleation, curettage, curettage with cryotherapy, resection. The patient was intubated via the orotracheal route and an excisional biopsy was performed. Deroofing of the lesion was done. Enucleation and curettage was done. Chemical cauterisation was done with Carnoy’s solution for 5 minutes, and later Bismuth iodoform paraffin pack was placed. Haemostasis was achieved and closure was done. On excision it showed islands of dense bony areas inside the lesion towards the lower cortex of the mandible. Microscopic examination revealed a proliferation of oval to spindle-shaped cells along with spherical mineralised ossicles resembling psammoma bodies which were basophilic at their centres and eosinophilic at the periphery with brush border. The supporting stroma also showed multiple sinusoidal spaces surrounded by many multinucleated giant cells. New bone formation was seen at the periphery. Follow-up was done after two months. (Figure 5), which showed bony healing at the lower border of mandible (Figure 6).

## Discussion

According to World Health Organisation (WHO), ossifying fibroma is classified into two types: the conventional/classic type and the aggressive type. The latter is further divided into aggressive trabecular and aggressive psammomatoid subtypes [9, 11]. Paul and Roman *et al* in 2005 classified it as conventional and JOF, of which juvenile has two variants, i.e., trabecular and psammomatoid [12]. PsJOF was initially described by Gogl in 1949 under the designation psammomatoid fibroma of the nose and paranasal sinuses [13]. Margo, in 1985 described PsJOF as a distinctive solitary fibro-osseous lesion of young individuals that affects the orbit and shows distinguishing histologic features. PsJOF was also reported under the designation juvenile active ossifying fibroma by Johnson *et al* [14]. In 1938; Benjamins reported the first case of an aggressive psammomatoid ossifying fibroma (APOF) of the frontal sinus [15]. Psammomatoid-type spherical ossicle structures are termed as psammoma-like bodies, which is derived from a Greek word ‘psammos’ meaning ‘sand’. Ultrastructurally, these psammoma-like bodies in PsJOF were found to possess a dark rim of crystals, from which small spicules and needle-like crystalloids project toward the periphery [16]. Similar bodies were also observed in our case. PsJOF, when it occurs in the jaws, is considered as a unique entity. Additionally, its association with ABC had been sparsely reported in the literature [6, 14, 17–19]. Rezwana Mohammed *et al* have reviewed cases of ABC occurring *de novo* and in pre-existing lesions from 1959 to 2011, which totalled 27 cases, of which three cases were of ossifying fibroma—two were the trabecular type of JOF and one conventional type, but there was no psammomatoid variant [20]. Makek reported that out of 69 cases of PsJOF, three cases showed ABC-like areas [10]. Aggressive growth has been reported in young patients as in our case [21]. Both these lesions, i.e., PsJOF and ABC, are aggressive and have a high recurrence rate, i.e., as high as (30–56% and 26–56%) respectively [14, 19]. Although the psammomatoid type is more frequently reported as compared to the trabecular type, i.e., 1:4 ratio, it is rarely reported in the jaws [6]. 90% of PsJOF occurs in the orofacial region, out of these 90% cases, 10% of cases occur in the mandible [22]. The current case was also observed in the mandible. Radiographically, as the lesion was multilocular mixed radioopaque and radiolucent lesion, chances of misdiagnosis are more, such as it being odontogenic tumours like ameloblastoma, non-odontogenic tumours like fibrous dysplasia, ossifying fibroma, ameloblastoma, ossifying fibroma, but in our case as the patient age was seven years which is very young, ameloblastoma and ossifying fibroma were ruled out and the diagnosis went in favour of JOF. Cherubism was ruled out because of the unilateral occurence of the lesion. Ameloblastoma, on the other hand, is multilocular radiographically with a characteristic of ‘soap bubble–like’ appearance [23], and the overall configuration of the JPOF is more rounded than the typically fusiform outline of fibrous dysplasia [24]. These lesions exhibited multilocular radiolucencies showing a blown-out appearance diagnostic of ABC [25] JOF has been classified as a different disease because of its local aggressive behaviour and its tendency to predominantly occur in children and adolescents. JOF had the youngest average age (20.5 years), followed by OF (25.1 years) and FD (29.6 years) [26]. Pre-existing fibro-osseous lesions are usually missed out like in our case PsJOF was missed out. Histologically, there is cellular fibroblastic stroma containing spherical and curved ossicles. These ossicles are concentric and show brush border at periphery. They are referred to as psammoma-like bodies or psammomatoid ossicles. They are usually without osteoblastic rimming [27]. Similar features were also seen in our case.

Additionally, cytological diagnosis of ABC has rarely been reported in the dental literature. [27, 28]. It shows cytologically moderately cellular stroma with benign appearance of oval to spindle-shaped cell clusters interspersed with multinucleated giant cells. Similar findings were seen in our case. Histopathologically, this hybrid lesion of PsJOF with ABC shows highly cellular hyperchromatic spindle- shaped stroma with psammomatoid ossicles of varing size diagnostic of psammomatoid ossifying fibroma. It also shows dilated blood-filled spaces surrounded by multinucleated giant cells suggestive of ABC-like changes. Similar findings were observed in our present case. The prognosis of such a lesion is good if diagnosed and treated carefully as in our case, which showed good healing after two months of surgery.

## Conclusion

PsJOF is a fibro-osseous lesion which occurs rarely in the mandible and behaves aggressively when occurring in young patients and when in association with ABC. Early correct diagnosis and proper treatment is necessary. If left untreated, extension of the lesion into nasal, orbital, and cranial cavities is common. Pre-treatment biopsy and CT scan is necessary for proper diagnosis and treatment plan of the lesion. Due to the high recurrence rate, long-term follow-up is necessary.
